# Quality Evaluation of High-Polyphenol Vinegars Produced from Various Romanian Plum Cultivars

**DOI:** 10.3390/foods14183282

**Published:** 2025-09-22

**Authors:** Maria-Cristina Todașcă, Cătălina-Beatrice Poteraș, Teodora-Alexandra Iordache, Mihaela Tociu, Ștefan Theodor Tomas, Georgeta Ștefan, Fulvia-Ancuța Manolache

**Affiliations:** 1 Faculty of Chemical Engineering and Biotechnology, National University of Science and Technology Politehnica Bucharest, 1-7 Gh. Polizu Street, 011061 Bucharest, Romania; catalina.poteras@stud.chimie.upb.ro (C.-B.P.); mihaela.tociu@upb.ro (M.T.); stefan.tomas@upb.ro (Ș.T.T.); 2 National Research & Development Institute for Food Bioresources—IBA, 5 Ancuta Baneasa Street, 020323 Bucharest, Romania; teodora.manasia@bioresurse.ro; 3 Faculty of Veterinary Medicine, University of Agronomic Sciences and Veterinary Medicine, 105 Spl. Independentei, 050097 Bucharest, Romania; georgeta.stefan@fmvb.usamv.ro

**Keywords:** plum vinegar, fermentation, total polyphenol content, ATR-FTIR assay, NMR

## Abstract

Plum vinegar represents a functional food product valued for its potential health benefits, largely attributed to its polyphenolic content. This study investigates the quality parameters of vinegars produced from five Romanian plum varieties (Centenar, Agent, Andreea, Anna Spath and Romanian Vanata), focusing on their physicochemical and phenolic profiles. Analyses included pH, total acidity, colorimetric evaluation, total polyphenolic content (TPC), antioxidant activity, and ATR-FTIR and ^1^H-NMR spectroscopy. The vinegars exhibited pH values ranging from 3.2 to 3.7 and TPC values between 250 and 350 mg GAE/L during fermentation, with higher concentrations (up to 400 mg GAE/L) recorded post-aging. These findings support the potential of Romanian plum varieties as valuable raw materials for producing high-quality, polyphenol-rich fruit vinegars.

## 1. Introduction

Vinegar is an acetic product resulting from the fermentation of sugars contained in sweet fruits (plums) by yeasts, resulting in alcohol, followed by an oxidation step of ethanol to acetic acid by bacteria. Vinegar was/is used as a seasoning product in various dressings, sauces, ketchups and mayonnaise, mainly for salads, or since Hippocrates’ era, in medicinal aids, for example, in curing sores, injuries or for antibacterial purposes [1,2].

There are several types of raw materials used to produce vinegars: fruits, vegetables, wine or honey/whey (animal-based raw materials). Currently, fruit vinegar has emerged as a healthy beverage abundant in bioactive compounds (e.g., polyphenolic, organic acids, melanoidins, etc.) with potential benefits in treating inflammation, diabetes, microbial side-effects or cancer [3,4,5].

Fruit vinegars contain high concentrations of polyphenolic compounds, and their final quality depends mainly on the raw material used as a substrate, acidification process, fermentation and aging procedures employed during manufacturing [6]. For example, the total phenolic content and total flavonoid content measured for plum vinegar was 470.86 mg catechin/L, DPPH 0.302 g TE/mL, ABTS 0.538 g TE/mL [7], which scored better than grape, apple, fig or apricot vinegars. A study made on fruits from the Algarve region confirmed the previous results and showed that plum vinegar scores TPC 51.0 mg GAE/100 mL, TAC 0.302 µg TE/mL, which is similar to raspberry or fresh fig vinegars [8]. There are numerous studies which have focused on the antioxidant capacity of traditional and commercial vinegars [6] and of nutrients and bioactive components contained due to their potential as functionalized foods.

Plums, apart from being distributed worldwide, are renowned for their huge contribution to satisfying the nutritional requirements of human diet. Plums are rich in carbohydrates, vitamins, minerals (mainly potassium), flavonoids and phenolic acids, helping to treat cardiovascular diseases, diabetes, blood circulation disorders, gastrointestinal malfunctions, etc. [9]. At the same time, plums are rich in volatile compounds (a total of 737 volatiles) and have specific aromatic characteristics [10].

Romania is the second highest plum-producing country in the world. According to FAO, in 2023 Romania produced 645.090 tons of plums. In Romania, non-marketable plums (or other various fruits) are used to produce “tuica” or “palinca”, a distilled beverage obtained after the ethanolic fermentation of sugars. During the distillation, an essential part of plums, antioxidants and especially polyphenols, are damaged and lost in the process.

Consequently, the experiments performed in this article have been targeted to reveal the quality of plum vinegars based on their physicochemical features (pH, total acidity, color, total polyphenolic content and antioxidant activity), made from five different plum varieties cultivated in Romania.

So far, since no studies have been published on determining the total polyphenolic content of the small-scale production of plum vinegars, sampling was performed to establish the evolution of polyphenolic content during vinegar fermentation and during the maturation phase. Additionally, the main regions corresponding to the principal chemical compounds (water, organic acids, mostly acetic acid or alcohol) were marked by ATR-FTIR assessments and the general profile of plum vinegars was evaluated by ^1^H-NMR spectroscopy.

## 2. Materials and Methods

### 2.1. Samples Characterization

For this study, five varieties of plums (*Prunus domestica* L.) cultivated in Romania have been used: Centenar, Agent, Andreea, Anna Spath and Romanian Vanata.

The Centenar, Agent and Andreea varieties were cultivated and harvested by the Institute of Research and Development Pitesti-Maracineni, Romania, while Anna Spath and Romanian Vanata were cultivated and harvested in a private orchard in Arges County, Romania. The sugar content and detailed characteristics of the plum varieties are described in Table 1. Several literature reports describe the fruit characteristics of the Romanian plum varieties [11,12,13,14].

### 2.2. Pilot Scale Vinegar Samples Production

Two sets of plum vinegar samples have been produced. One set, consisting of six samples, was used to set up the optimal production inoculant (described in Table 2), and five samples were used to identify the differences generated by the plum variety (described in Table 3).

For the first set of samples, the six vinegar samples were produced at pilot scale using the two fermentation methods (alcoholic and acetic) of fresh plum fruits, enhanced by yeast and commercial vinegar addition. Briefly, 300 g of plums (pulps and peels, carefully washed) from three plum varieties (Centenar, Agent and Andreea) were mixed with 300 mL of purified water and 1 g of *S. cerevisiae* yeast. To assess the alcoholic fermentation, the samples were maintained at room temperature, away from direct sunlight. Following the alcoholic fermentation, 5 mL of commercial apple vinegar or Modena balsamic vinegar, were added to each glass and left for acetic acid fermentation development at room temperature (25 °C), covered with textile material. Prior to the analysis, the vinegar samples were separated through filtration from the course deposit and then stored in hermetic vials. In Table 2, all the details regarding the raw materials are included.

These samples were used to establish optimal conditions for plum vinegar production. The optimal conditions set in place were used to obtain plum vinegars from the second set of samples, as described in Table 3.

The second set of samples consisted of vinegars obtained from five plum varieties, at pilot scale: Centenar (P1Cen), Agent (P2Ag), Andreea (P3An), Anna Spath (P4AS) and Romanian Vanata (P5RV).

An amount of 300 g of fruit was used from each plum variety for plum vinegar production in the optimized condition. Vinegar production was carried out in glass vessels maintained at room temperature (25 ± 2 °C), protected from direct sunlight, and covered with sterile gauze to ensure gas exchange while preventing contamination. Upon completion of alcoholic fermentation, 5 mL of commercial apple vinegar were added to each sample to initiate acetic acid fermentation. The samples were manually agitated daily to enhance oxygen transfer and homogenization. Fermentation proceeded for 43 days, until the complete conversion of ethanol to acetic acid. Subsequently, the vinegar was separated from coarse deposits by filtration and transferred into hermetically sealed vials. Approximately 450 mL of vinegar were obtained per sample and stored at room temperature in the dark until further analysis.

### 2.3. The Physicochemical Parameters of Plum Vinegars

#### 2.3.1. Color Measurement

**Figure 1:**
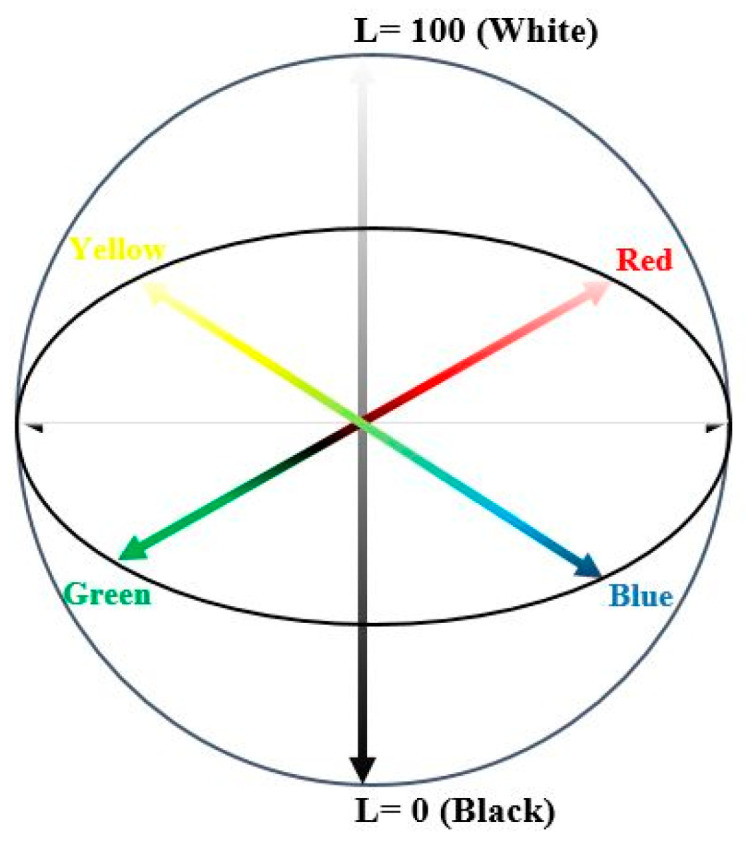
The diagram of the CIELAB color space parameters, L*, a* and b* (Source: [15]).

The plum vinegar color was determined using Konica Minolta colorimeter, equipped with color Data Software CM-S100w, SpectraMagicTM NX (Konica Minolta Sensing, Inc., Osaka, Japan). For the liquid samples analysis, plastic cells (10 mm) were filled, without air bubbles. The samples were placed in the measurement zone. L* (lightness), a* and b* chromaticity parameters were recorded and summed up, after three replicates of each sample [7]. The diagram of the CIELAB color space parameters, L*, a* and b* is described in Figure 1.

#### 2.3.2. pH Analysis

The pH analysis was conducted using a stationary pH-meter PH80 + DHS (Dostmann Electronic GmbH, Wertheim Reicholzheim, Germany) calibrated with the appropriate buffer solutions.

### 2.4. The Bioactive Compounds Present in Fruit (Plum) Vinegars

#### 2.4.1. Total Polyphenol Content

Folin–Ciocalteu (FC) assay was employed to assess the total concentration of polyphenols in plum vinegars. The method consists of the reduction in FC reagent in the presence of polyphenol compounds, part of the vinegar solution, through color modification, in a basic medium. An aliquot of each of the samples, 0.1 mL, was mixed with 3.9 mL of ultra-purified water. An amount of 0.5 mL of FC was then added. To obtain the basic medium, 0.5 mL of sodium carbonate, 20% was used. The samples were allowed to complete the reaction, in the dark, for 1 h. The absorbance was measured at 765 nm, using a Mettler Toledo spectrophotometer UV5 (Mettler Toledo International Inc., Greifensee, Switzerland). Gallic acid, 1 mg/L, served for mapping the calibration curve. Five serial dilutions were performed and analyzed according to the sample procedure [16]. The regression equation y = 106.33x, R^2^ = 0.998 assisted the data calculation.

#### 2.4.2. Antioxidant Activity

Antioxidant activity was measured using DPPH assay, following Artem V. et al. (2021) and Ali Z. et al. (2019) [17,18], with minor adjustments. In total, 100 μL of each sample was diluted 1:10 with methanol and then 200 μL solution was mixed with 3800 μL of 0.1 mM DPPH working solution (DPPH and methanol) in a 5 mL volumetric flask. After vertexing at 1600 rpm for 1 min, the solution was incubated for 30 min in the dark. The absorbance of the test sample was measured at 517 nm using a spectrometer (Mettler Toledo UV 5). Antioxidant activity was calculated using a calibration curve (0.2–1 mmol/L) obtained with Trolox (R^2^ = 0.9958). The results were expressed in mmol Trolox equivalent/L of plum vinegar.

#### 2.4.3. ATR-FTIR Analysis

The spectral data were acquired on a Bruker FTIR spectrophotometer (INVENIO S, Bruker, Karlsruhe, Germany), equipped with a detachable multi-reflection attenuated total reflectance (ATR accessory), ZnSe crystal. The samples’ measurements were performed in the 4000–400 cm^−1^ range at 32 scans with a resolution of 4 cm^−1^. Before the sample analysis, a background spectrum was recorded on the empty ATR, using the same acquisition parameters. The samples were simply placed in drops over the ATR crystal, using a micropipette (20–200 µL). Every sample acquisition was closely followed by thorough clean-up of the crystal to prevent contamination. The spectra of each sample was double collected and sample information was further gathered [19,20].

#### 2.4.4. ^1^H-NMR Spectroscopy

The spectral data were acquired on a Bruker Avance III 400 spectrometer (Bruker, Karlsruhe, Germany), operating at 9.4 Tesla, corresponding to the resonance frequency of 400.13 MHz for the ^1^H nucleus, equipped with a direct detection four nuclei probe head and field gradients on z axis. Parameters for ^1^H-NMR with water suppression from Bruker TopSpin were used for spectral acquisition (noesygpp1d). The acquisition parameters used were 90° high-power pulse, 2.55 s acquisition times, 6.4 KHz spectral window, 16 scans, and fid resolution 0.39 Hz. The average acquisition time of the ^1^H-NMR spectra was approximately 2 min. Sample preparation was carried out directly on the plum vinegar sample, without any prior derivatization, allowing analysis in its native form. A volume of 0.9 mL plum vinegar was mixed with 0.1 mL of deuterated water (D_2_O) with 10% TSP as internal standard, and the resulting mixture was transferred into 5 mm NMR tubes (Wilmad 507) for spectral acquisition. All spectra have been processed with TopSpin 4.0.7. software. The chemical shift was measured based on the internal standard TSP (trimethylsilyl propionate, sodium salt).

#### 2.4.5. Statistical and Chemometric Analysis

In food science, analyzing data through statistical procedures is necessary for the planning, analysis and interpretation of experimental work. This study includes surveys of the total polyphenolic compounds, and changes in the chemical composition during the fermentation processes. Minitab Statistical Software (version 21.4.3, Minitab, LLC, State College, PA, USA) and MS Office 365-Excel software were used for statistical analysis. Analysis of variance (ANOVA) with a Tukey post hoc test was used to evaluate differences between means, with a 95% confidence. The presence of different superscript letters in tables indicates significant differences (*p*-value < 0.05). The Pearson correlation index was utilized to examine linear correlations between the total phenolic compounds of the vinegars with a minimum level of significance of *p*-value < 0.05.

The chemometric analysis of the data was performed using the XLStat 2023 software (developed by Addinsoft, Paris, France). Principal Component Analysis (PCA) was performed on the concentration of the main components of plum vinegar, quantified based on NMR data.

## 3. Results and Discussion

### 3.1. Color Measurement

**Figure 2:**
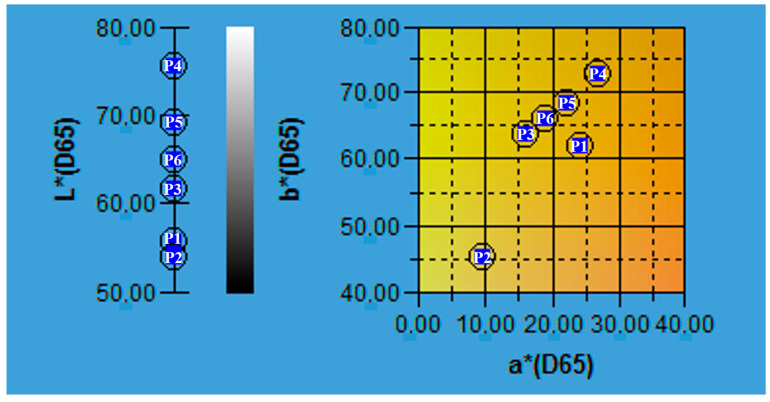
The samples of vinegar on the CIELAB map of colors (where P1—P1ACen, P2—P1BCen, P3—P2AAg, P4—P2BAg, P5—P3AAn and P6—P3BAn).

Since consumer acceptance is guided by the sensorial attributes of products, an important aspect for vinegar is color. For the six plum vinegar samples produced at pilot scale to determine the optimal production conditions, the color analysis showed values of 54.03–75.31 for L* (lightness), 9.76–26.84 for a* (redness) and 45.12–72.73 for b* (yellowness) [9]. The results are shown in Figure 2.

Based on the color analysis, the lightest sample proved to be P2BAg, whereas the darkest, P1BCen and P1ACen. The color intensity is correlated with the high flavonoids and anthocyanins content of plum peels which were extracted during fermentation steps of the vinegar production [10]. These results show that the inoculant for pilot scale preparation of the plum vinegar samples has no important influence on the quality of the final product, when the flavonoids and anthocyanins content are in discussion.

The main variable that influences the final color of the plum vinegar is the fruit color in accordance with the plum variety used in the vinegar production process. The initial color of the fruit (described in Table 1, varies from red to violet and blue) is reflected in the color of the plum vinegar obtained, since during the fermentation process the pigment vacuoles from plum’s exocarp are broken and the anthocyanins are released and extracted in the low alcoholic acid medium.

### 3.2. Physicochemical Parameters of Plum Vinegars

The content of organic acids is strongly linked to the organoleptic properties of plum vinegars. The plum vinegars’ acidity, expressed as acetic acid (g/100 mL), varies between 0.1 and 3.4 g/100 mL; meanwhile, pH ranges from 3.2 to 3.68, as presented in Table 4 [21]. Usually, the pH of fruit vinegars shall range within 2.4–3.9 [3] and the degree of acidity is no less than 4% acetic acid (*w*/*v*), according to international regulation standards [22]. Still, as was previously reported by Ozturk et al., 2015 [7], the majority of traditional samples (e.g., grape, apple, apricot, pomegranate, apple–lemon vinegars), were besides the imposed limit. The same principle extends to the plum vinegar samples obtained in this experimental stage, whose maximum acidity is less than 3.4 g/100 mL acetic acid.

**Figure 3:**
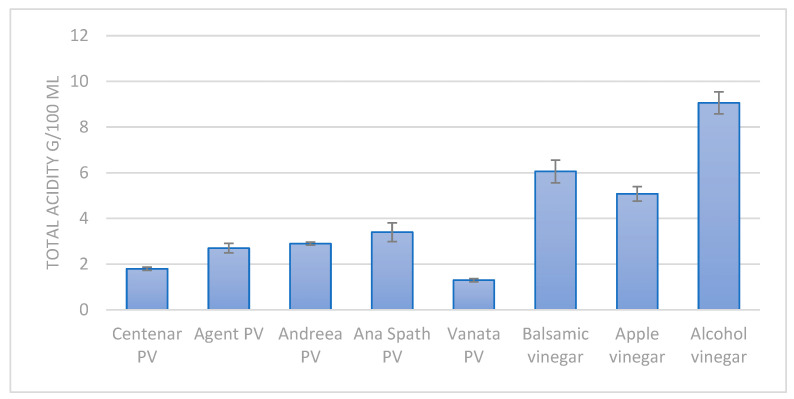
The total acidity of the plum vinegar (PV) samples in comparison with commercially available vinegars.

Compared with commercially available vinegars, the results show a lower acidity in the plum vinegar samples (Figure 3) in comparison with apple vinegar or balsamic vinegar. The low acidity of the plum vinegars is generated by the initial lower acidity of the fruits and the lower sugar content of the plums in comparison with apples. The mean sugar content in plums is 9 ± 1.5 g/100 g of fruit, while the mean sugar content for apples is 10 ± 1.3 g/100 g of fruit.

### 3.3. Total Polyphenolic Content (TPC)

The antioxidant activity of fruit-derived products is attributed to the presence of bioactive compounds (e.g., polyphenols, ascorbic acid, carotenoids) coming from the raw materials. According to a study performed by Shahidi et al., 2008 [23], among the eight fruit/plant-based vinegars targeted (plum, rice, apple, apple cider, etc.), in view of superior functional potential, plum vinegar recorded the highest concentration of phenolics. Lin et al., 2023 [10], studied the antioxidant activity of five plum cultivars from China, Sichuan region, since plum is an essential plant crop, recently researched for its nutraceutical potential. The concentration of TPC determined in the analyzed samples was between 120 and 320 mg GAE/100 g. Previously reported literature data confirmed values ranging from 100 to 372 mg GAE/100 g in different plum fruits grown in different regions of the world.

**Figure 4:**
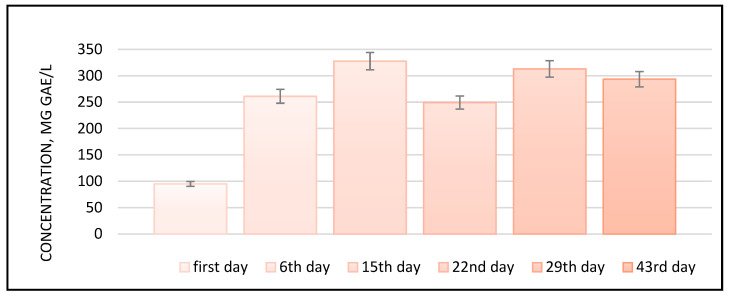
Evolution of total polyphenolic compounds in the plum vinegar sample P1ACen during the fermentation processes; *p* < 0.05.

Following the evolution of the TPC of the plum vinegar samples produced at pilot scale during this study, similar results have been outlined (e.g., a maximum of 350 mg GAE/L for P2Ag at the end of the alcoholic fermentation process). For the rest of the samples, the TPC average ranked 250–300 mg GAE/L, with small variations, into the fermentation stage. The variation in the TPC during the fermentation process is shown in Figure 4; it uses the Centenar variety as an example.

Addressing the maturation phase of vinegars, it is worth mentioning that the variation in the polyphenols content is small for all five varieties of plum vinegars, ranging from 360 to 400 mg GAE/L.

For statistical analysis, the normality test of the samples must be checked. Just P1ACen and P1BCen samples have *p*-value < 0.05, which means that the null hypothesis is accepted (The statistical population from which the sample comes follows a normal distribution).

For ANOVA, the test for equal variances must also be checked, where the results of the normality test, depending on the *p*-value, will be specified. For all pairs of samples, *p <* 0.05, which means that the null hypothesis is accepted, i.e., the variance of our system corresponds to that of the population it refers to, proving that the results are relevant.

An evaluation of the TPC was made in the case of the six vinegar samples obtained for the optimization of the production procedure. In Table 5 the TPC is summarized, and it can be noticed that the vinegar inoculant has no important influence on the amounts of polyphenol obtained in the final product (plum vinegar). A slightly larger difference was noticed between the samples obtained from the Agent variety (P2AAg and P2BAg). This difference was generated by fruit characteristics and the low sugar concentration which was responsible for the delayed extraction of the anthocyanins from the pigment vacuoles of the exocarp, due to the low amount of acetic acid developed in the fermentation process and due to the thick and firm plum skin. Therefore, we decided to use apple vinegar as the inoculant in the process of obtaining plum vinegar.

**Figure 5:**
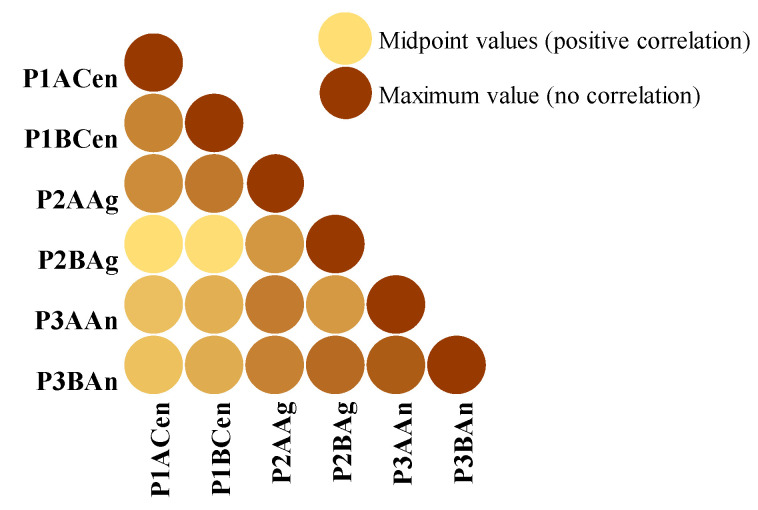
Pearson correlation coefficient between the six samples used for process optimization, using conditional formatting.

The Pearson correlation coefficient measuring the linear correlation between the six sample groups used for process optimization has indicated a positive correlation among them. This result shows that the TPC is increasing simultaneously for all six samples used for establishing the optimal conditions for plum vinegar processing, as presented in Figure 5. Therefore, the inoculant used in the process has no important influence on the quality of the final product.

**Figure 6:**
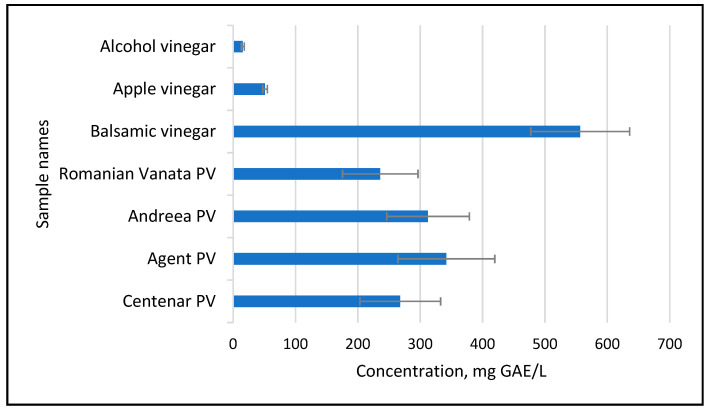
TPC of the five plum variety vinegar (PV) samples in comparison with commercial vinegars.

Considering the medium values of the TPC in the five varieties of plum used for plum vinegar preparation, an evaluation was carried out in comparison with commercially available vinegar samples. As shown in Figure 6, the amount of TPC obtained in the plum vinegars is comparable with balsamic vinegar and higher than the TPC amount available in apple vinegars or in vinegar obtained from fermentation of food-grade alcohol.

These results are in line with other studies made on plum vinegar originating from Portugal or China [8,10]. The high content of polyphenols present in the plum peel (the TPC is 7158–14,770 mg/kg FW [12]) is extracted in the vinegar during the fermentation process when the pigment vacuoles in the exocarp are broken. This makes plum vinegar richer in polyphenols in comparison with other fruit vinegars, like apricot, apples and even white grapes.

### 3.4. Antioxidant Activity

An evaluation of the antioxidant activity was made in the case of the six vinegar samples obtained for the optimization of the plum vinegar production procedure. The DPPH radical scavenging activity assay was used to establish the antioxidant capacity. The plum vinegar samples exhibit a good antioxidant activity, which is corelated with the total polyphenol content.

**Figure 7:**
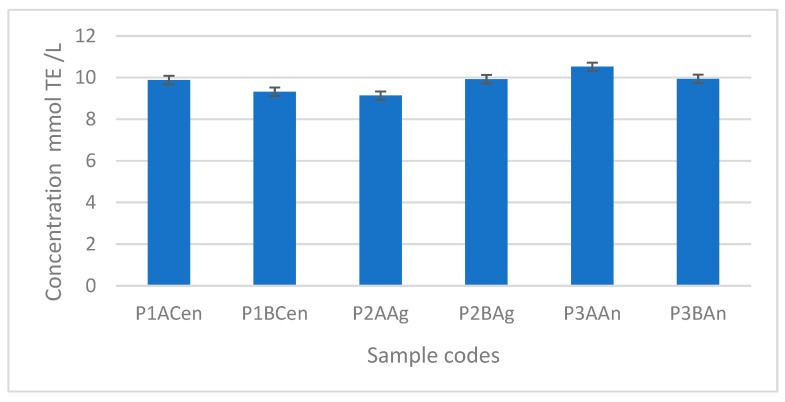
Antioxidant activity of the plum vinegar samples expressed in mmol Trolox equivalent (TE)/L.

Compared with the literature data, the antioxidant activity of the obtained plum vinegar, presented in Figure 7, is comparable or higher than other fruit vinegars (for instance apple vinegar), and much higher in comparison with fresh or maturated cereal-based vinegars [24,25]. The highest antioxidant activity was obtained for the vinegar sample obtained from the Andreea plum variety, with no noticeable difference when apple vinegar or balsamic vinegar was used as the inoculant.

Based on the results obtained with the purpose of establishing the optimal processing conditions for plum vinegars using the six samples of plum vinegar attained with two types of inoculants, apple and balsamic vinegar, we concluded that using apple vinegar offers optimal conditions for obtaining high-quality plum vinegars.

The antioxidant activity for all plum vinegars obtained in optimal conditions ranged between 10.9 and 8.6 mmol TE/L.

### 3.5. ATR-FTIR Assay

**Figure 8:**
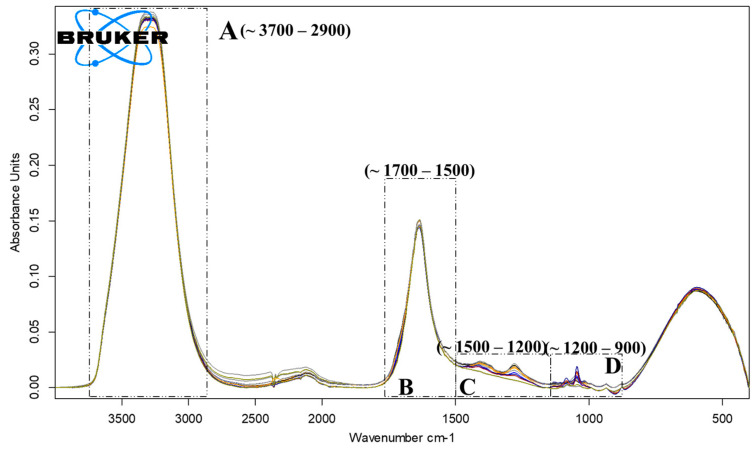
ATR-FTIR spectrum of Centenar samples (12 samples) during the fermentation stages, highlighting the regions with the most significant changes.

ATR-FTIR analysis has pointed out the presence of four areas, A, B, C and D, representative for the principal chemical compounds found in vinegar [19]. The zone A, 3700–2900 cm^−1^ is attributed to the specific stretching of the O-H and C-H bonds part of water and acetic acid molecules [20], see Figure 8 and the corresponding attribution in Table 6.

**Figure 9:**
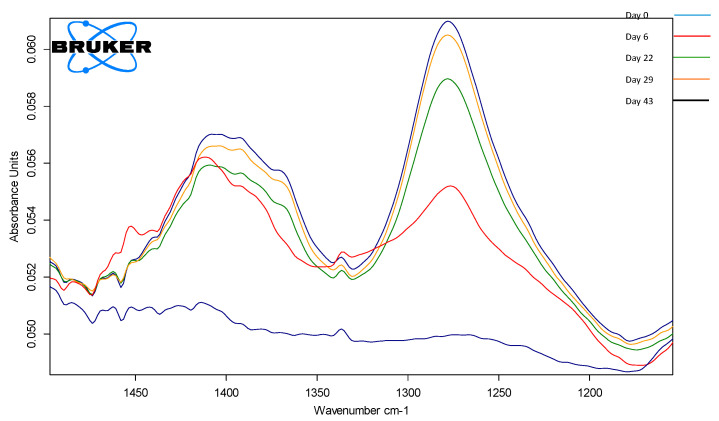
ATR-FTIR spectrum of P1BCen samples during the fermentation stages, highlighting the region 1500–1200 cm^−1^.

Using the absorption band between 1500 and 1200 cm^−1^, evaluation of the production conditions could be made since the changes in this spectral area are influenced by the processing parameters. In Figure 9, the compositional changes during the fermentation process are visible, and using a calibration curve enables the quantification of the acetic acid during formation.

FT-IR allows real-time visualization of acetic acid formation, helping the process engineers to take decisions regarding processing steps, and especially deciding on the end of the fermentation process.

### 3.6. ^1^H-NMR Analysis

The ^1^H-NMR general profile of the plum vinegars obtained helps us to identify and quantify a total of 10 individual chemical compounds (methanol, polyols, carboxylic acids) apart from the acetic acid. The minor components support the quality claim of the obtained plum vinegars and help to differentiate the vinegars in accordance with the plum variety and to assess its quality and authenticity.

**Figure 10:**
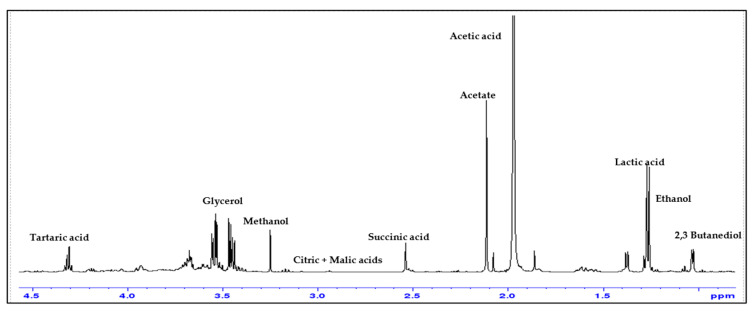
Area 4.5–0 ppm of the ^1^H-NMR spectrum of P4AAS plum vinegar sample, highlighting the peaks used as markers for the main components.

In Figure 10, the Anna Spath plum vinegar NMR spectrum is presented for exemplification and the marker signals for the main components are assessed [26,27].

**Figure 11:**
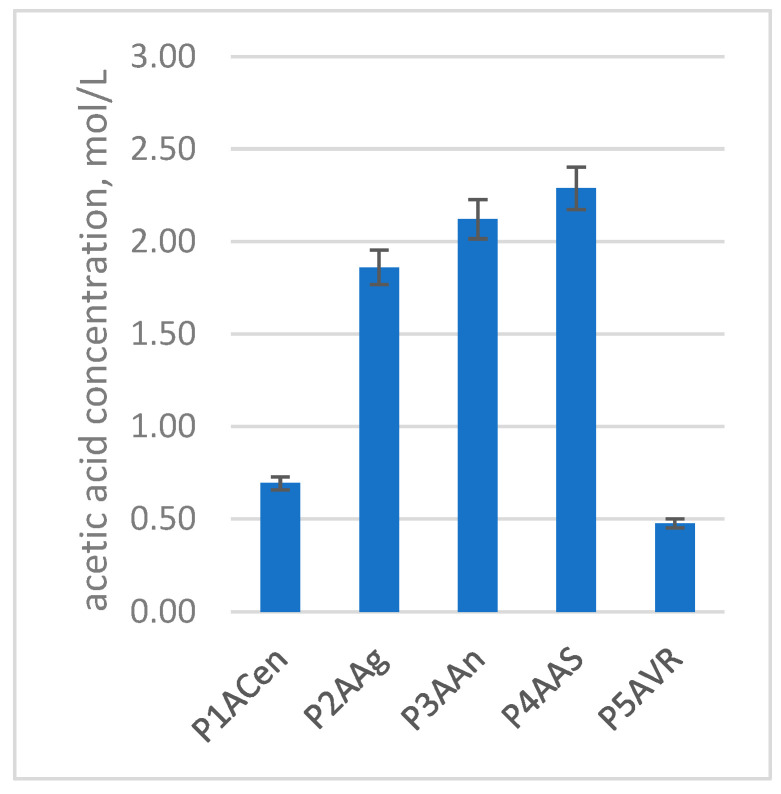
Concentration of acetic acid (mol/L) in the plum vinegars obtained from five different varieties of plum.

There are three levels of concentrations for the main compounds that can be identified using ^1^H-NMR spectroscopy: the highest level of concentration is water and acetic acid; on the second level of concentrations we have the alcohols (ethanol, methanol, 2,3-butanediol, glycerol), the carboxylic acids (lactic, succinic, citric, malic and tartaric acids except acetic acid) and acetates; and on the third concentration level we have the polyphenols. The polyphenols are the only ones which cannot be quantified from NMR due to the reduced concentration. The amount of the main compounds quantified in the obtained plum vinegar samples, by means of NMR spectroscopy, are shown in Figure 11.

As shown in Figure 11 and Table 7, the main compositional differences between the plum vinegar samples are determined by the plum variety used in the process. When considering the acetic acid composition, the best results were obtained from the Anna Spath, Andreea and Agent plum varieties, and the least successful type was Romanian Vanata. Therefore, selecting the plum variety ensures successful preparation of high-quality plum vinegars.

The initial composition of the fruit is an important factor in the final composition of the plum vinegar. As can be noticed in Figure 11 and Table 7, the main compounds identified and quantified in the plum vinegars have large variations from one variety to another. The glycerin content reaches 405 mmol/L in the Agent variety, while the Anna Spath, Andreea and Centenar varieties have values in the range 110–161 mmol/L. Glycerin is formed during alcoholic fermentation in secondary reaction, and its amount is influenced by the fermentation conditions. The yeasts tend to produce more glycerin in stressful conditions (low or high pH, high alcohol level, etc.) to protect themselves. Glycerin plays an important role in the taste of the final product, contributing to a more pleasant (velvet-like) taste of the plum vinegar.

The number of carboxylic acids, except acetic acid, are higher in the Anna Spath and Agent varieties, while the lowest amount is measured in the Centenar and Romanian Vanata varieties. As shown in Figure 11, the highest concentration of acetic acid is obtained for Anna Spath, followed by the Agent and Andreea varieties. These results corelate with the higher sugar content of the plum varieties used. Therefore, for plum vinegar production, those varieties should be the first option of choosing.

**Figure 12:**
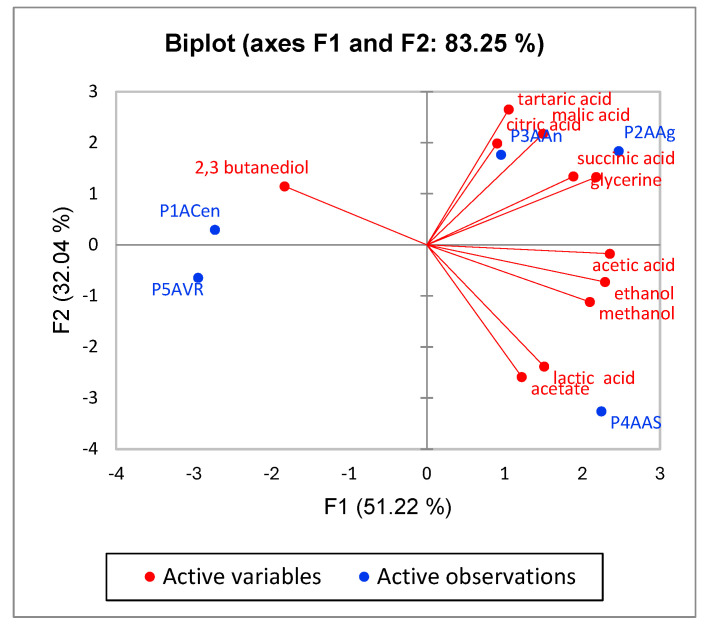
Principal Component Analysis for the five plum vinegars obtained from different plum varieties.

The concentration of the main components from the plum vinegars obtained by NMR analysis have been analyzed by means of Principal Component Analysis (PCA). The results presented in Figure 12 show that the plum vinegar obtained from the Anna Spath variety (P4AAS) has a distinctive composition when compared with the other four varieties, while the plum vinegar obtained from the Agent and Andreea plum varieties have a similar composition, since P2AAg and P2AAn tend to cluster in the same region. The results confirm the conclusions obtained with the classical analysis (pH, TPC, antioxidant capacity) and direct the selection decision of plums intended for producing high phenolic concentration plum vinegar towards the Anna Spath, Andreea and Agent varieties.

## 4. Conclusions

The present study aims at developing new high polyphenolic vinegars starting from five plum varieties cultivated in Romania. The physicochemical analysis, total polyphenol content (GAE/L), as well as the presence of main chemical compounds using ATR-FTIR approach and ^1^H-NMR analysis support the quality claim. The total acidity values (g/100 g) revealed one distinctive characteristic from the conventional vinegars (values smaller than 4% acetic acid, as requested by the legislation standards), and have highlighted that these vinegars are not as common. The best plum varieties in terms of acetic acid formation in the final product are Anna Spath, Andreea and Agent. The high values of polyphenolic content found in samples P2AAg and P4AAS indicated the superiority of Agent and Anna Spath plum varieties in the production of high-quality plum vinegars. Therefore, plum variety plays an important role in the development of high polyphenolic vinegars comparable to balsamic vinegars.

The optimal processing conditions have been established and the pilot-scale-obtained plum vinegars exhibited superior quality in comparison with other fruit vinegars. Less commercially attractive fruits and fruit by-products, which regularly become waste, are now used as important raw material capable of producing high-quality vinegars. The high values of phenolic content found in the obtained samples pointed to the superiority of these, compared with recognized high-quality commercial vinegars.

## Figures and Tables

**Table 1:** Detailed characteristics of the plum varieties used in this study.

	Plum Variety	Fruit Weight, g	Karnel Weight, g/1 kg of Plums	Sugar Content, %	Fruit Characteristics
1.	Centenar	30 ± 2.41	41.6 ± 1.4	8.9–10.89	Ovoid shaped fruit, with a brown-red-violet color
2.	Agent	33 ± 5.1	35.8 ± 2.1	9.8–10.9	Elongated spherical shaped fruit, with a red color
3.	Andreea	40 ± 5.1	35.9 ± 3.3	8.18–11.9	Spherical shaped fruit, with a red-violet color
4.	Anna Spath	41 ± 6.0	35.7 ± 3.2	10–12.89	Oval shaped fruit, with a violet-blue color
5.	Romanian Vanata	22 ± 4.8	35.5 ± 2.5	8.4–9.9	Elliptical shaped fruit, with a blue color

**Table 2:** Detailed information about the raw materials that served plum vinegar production.

No.	Plum Varieties Details	Sample Codes	Ingredients				
			Plums, g	Purified Water, mL	*S. cerevisiae* Yeast, g	Commercial Apple Vinegar, mL	Commercial Modena Balsamic Vinegar, mL
1.	Centenar	P1ACen	300	300	1	5	0
2.		P1BCen				0	5
3.	Agent	P2AAg				5	0
4.		P2BAg				0	5
5.	Andreea	P3AAn				5	0
6.		P3BAn				0	5

**Table 3:** Detailed information about the raw materials that served plum vinegar optimal production.

No.	Plum Variety	Sample Code	Ingredients			
			Plums, g	Purified Water, mL	*S. cerevisiae* Yeast, g	Commercial Apple Vinegar, mL
1.	Centenar	P1ACen	300	300	1	
2.	Agent	P2AAg				5
3.	Andreea	P3AAn				
4.	Anna Spath	P4AAS				
5.	Romanian Vanata	P5ARV				

**Table 4:** The summarization of physicochemical characteristics determined for the vinegar samples.

No.	Sample Codes	pH	Total Acidity ± Stdev *, g/100 mL
1.	P1ACen	3.48	1.8 ± 0.07
2.	P2AAg	3.29	2.7 ± 0.21
3.	P3AAn	3.68	2.9 ± 0.07
4.	P4AAS	3.36	3.4 ± 0.42
5.	P5AVR	3.20	1.3 ± 0.07

* stdev—standard deviation of three measurements.

**Table 5:** The summarization of total phenolic compounds concentration determined for the vinegar samples after 43 days of fermentation.

Crt.	Sample Codes	Total Polyphenolic Content ^1^ ± Stdev, g/mL
1.	P1ACen	260.22 ± 61.2 ^A,a^
2.	P1BCen	267.89 ± 64.68 ^AB,a^
3.	P2AAg	341.85 ± 77.66 ^AB,b^
4.	P2BAg	282.78 ± 62.71 ^AB,a^
5.	P3AAn	316.93 ± 81.15 ^B,a^
6.	P3BAn	312.52 ± 66.20 ^B,a^

^1^ stdev—standard deviation of three measurements, a, b, A and B—are used in post-hoc analysis to indicate grouping information; groups with different letters are significantly different at confidence level *p* < 0.05.

**Table 6:** The specific spectral regions of vinegar’s main compounds.

No.	Areas, cm^−1^ (FTIR Data)	Chemical Compounds’ Group
1.	A: 3700–2900	stretching vibration of O-H groups (water and other O-H groups)
2.	B: 1700–1500	stretching vibration of C=O groups, from carboxylic acids (mainly acetic acid) or aldehydes
3.	C: 1500–1200	stretching vibrations of C-O bond and, respectively, the C-O-H shearing in the plane.
4.	D: 1200–900	bending vibrations of carboxylic acids, aldehydes, esters, ethers, alcohols and phenols and some nitrogen compounds present in vinegar

**Table 7:** Concentration of the main components (mmol/L) in the plum vinegars obtained from five different varieties of plum.

Sample Codes	2,3 Butanediol	Ethanol	Lactic Acid	Acetate	Succinic Acid	Malic Acid	Citric Acid	Methanol	Glycerol	Tartaric Acid
P1ACen	4.62	1.40	30.07	3.68	8.33	2.11	0.00	2.77	110.30	39.33
P2AAg	4.19	3.49	47.45	45.67	26.57	83.34	9.06	19.63	405.17	94.95
P3AAn	4.86	3.50	23.88	3.91	27.24	31.72	31.10	13.88	154.47	55.63
P4AAS	2.56	4.24	165.92	122.23	18.72	0.00	0.00	23.76	161.24	0.00
P5AVR	6.96	2.20	6.44	43.72	8.05	0.00	0.00	12.22	19.84	0.00
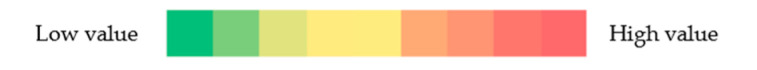										

## Data Availability

The data presented in this study are available on request from the corresponding author.
